# Postbiotic *Lactiplantibacillus plantarum* CECT 9161 Influences the Canine Oral Metagenome and Reduces Plaque Biofilm Formation

**DOI:** 10.3390/ani15111615

**Published:** 2025-05-30

**Authors:** Adrián Florit-Ruiz, Laura Rago, Antonia Rojas, Bellahanum Guzelkhanova, Adrià Pont-Beltran, Araceli Lamelas, María Carmen Solaz-Fuster, Juan F. Martinez-Blanch, María Enrique López, Guillermo García-Lainez, Bob T. Rosier, Richard Day, Teresa Rubio, Rhiannon Batchelor, Sophie L. Nixon

**Affiliations:** 1 ADM R&D Health & Wellness, 46980 Paterna, Spain; 2 Fundació Fisabio, 46020 València, Spain; 3 ADM R&D Health & Wellness, Somerset TA13 5JH, UK; 4 Ontario Nutri Lab, Fergus, ON N1M 2W4, Canada

**Keywords:** oral health, dog, microbiome, *Lactiplantibacillus plantarum*, plaque, oral biofilm

## Abstract

Periodontal diseases are highly prevalent in dogs and intricately interconnected with the composition and functional attributes of the oral microbiota. The demand for non-invasive interventions to support oral health presents an opportunity for functional ingredients. The novel postbiotic heat-treated (HT) *Lactiplantibacillus plantarum* CECT 9161 inhibited growth and biofilm formation of oral microorganisms in vitro. The in vitro growth of saliva-derived biofilms was also inhibited and revealed microbiome modulation. Two doses of the postbiotic (LOW: 5 mg dog/day, HIGH: 25 mg/dog/day) were assessed in a placebo-controlled, double-blinded, 57-day clinical trial involving 60 dogs. Associations were found between the postbiotic, reduced plaque formation, and modulation of the oral microbiome, including increased abundance of genes involved in denitrification, heme and catechol biosynthesis, and oxidative stress reduction. The results suggest that HT *Lactiplantibacillus plantarum* CECT 9161 may support oral health in dogs by modifying the microbiome of supragingival plaque and reducing plaque formation.

## Simple Summary

Maintaining good oral health is important to the wellbeing of dogs. Build-up of dental plaque is associated with poor oral health and can ultimately lead to tooth loss. Toothbrushing is one of the most effective ways of controlling dental plaque, but regular toothbrushing can be challenging to implement. Postbiotics have the potential to reduce plaque build-up without toothbrushing but are under-researched in dogs. The aim of the present study was to use preclinical screening to identify a candidate postbiotic to reduce plaque build-up in dogs, and to test this postbiotic in a clinical study. The novel postbiotic heat-treated (HT) *Lactiplantibacillus plantarum* CECT 9161 was identified in the preclinical pipeline for its ability to reduce the growth of oral bacteria and was taken forward to a clinical study in dogs. In the clinical study, the high-dose postbiotic was shown to reduce plaque build-up by over 10% in dogs when applied once daily on kibble, and the low-dose postbiotic was associated with a non-significant tendency towards a 17% reduction in plaque formation. The postbiotic was also associated with changes in the oral microbiome that might be beneficial to health. Taken together, these findings provide the first evidence that the novel postbiotic HT *Lactiplantibacillus plantarum* CECT 9161 may support oral health in dogs by reducing plaque formation when applied on kibble.

## 1. Introduction

The oral microbiome plays a key role in health and disease in both humans and dogs [1,2,3]. Periodontal diseases are one of the most common diseases seen in dogs [4], with the prevalence reported to range from 20 to 84% and some evidence that this is higher for wet food than dry food [5,6,7,8]. Periodontal diseases include gingivitis and periodontitis [9]. Gingivitis describes an inflammation of the gingiva and can be reversed through intervention, including dental plaque removal by oral hygiene. Periodontitis is the irreversible progression of gingival inflammation, characterised by the destruction of the tissues that support the tooth, leading to attachment loss due to breakdown of the periodontal ligament [9]. Gingivitis is initiated by the build-up of plaque on the tooth surface as a host response to accumulated bacteria. Long or repeated episodes of gingivitis increase the risk of periodontitis associated with a dysbiotic subgingival microbiota [9]. When canine dental plaque is formed, early colonizers include *Neisseria weaveri* and *Neisseria zoodegmatis*, which facilitate the attachment and growth of other bacterial species [10]. The resultant biofilm continues to develop into mature plaque over time through an ordered process of bacterial succession [10]. The biofilm slowly mineralises and starts developing into calculus within 48 h. The formation itself is not pathogenic; however, as plaque biofilms mature, they become thicker and oxygen levels decrease, favouring anaerobic microbial species associated with poor oral health [10]. Anaerobic pathobionts in dental plaque can cause damage through the secretion of proteinases, siderophores, toxins, and pro-inflammatory molecules, triggering a host immune response and dysregulated inflammatory cascade leading to gingivitis and, in some cases, periodontitis [10]. The “keystone pathogen” hypothesis proposes that certain oral pathogens can, even at low abundance, orchestrate inflammatory disease by triggering a cascade of changes in the oral microbiome resulting in a dysbiotic state [11].

The canine oral microbiome has been shown to be widely divergent from that of the human microbiome, with only 16.4% of shared taxa [12]. Distinctive bacterial communities have been associated with healthy and diseased periodontal tissue. *Porphyromonas* species have been isolated from the periodontal pockets of dogs with periodontal disease [13], and a recent study indicated an increase in bacteria belonging to the Proteobacteria phylum and a decrease in *Actinobacteria*, *Firmicutes*, and *Bacteroidetes* phyla from dogs with periodontal disease compared with healthy dogs [14], although this study only assessed cultivable bacteria so may be prone to bias. Differences in relative abundance of bacterial species are associated with the development and progression of periodontitis, rather than the simple presence or absence of species [14,15]. The presence of inflammation and the formation of a periodontal pocket change the environment, giving a selective advantage to disease-associated bacteria, while decreasing the growth of health-associated species.

Current options to prevent periodontal disease usually focus on the mechanical removal of plaque such as tooth brushing, which is considered the most effective means of preventing oral disease [16]. Low levels of owner compliance or non-acceptance by dogs leads to low levels of interventions in the pet population [17,18]. Useful additions to tooth brushing include dental chews, dental diets, and chew toys [17,18,19]. Trials investigating the use of pre-, pro-, and postbiotics (collectively “biotics”) in periodontal disease have recently gained interest as non-invasive methods of influencing oral health. Whilst the concept of probiotics and prebiotics is widely understood as live microorganisms that confer a health benefit, and a nonviable food component that confers a health benefit associated with microbiota modulation, respectively, the notion of postbiotics is more recent. The International Scientific Association of Probiotics and Prebiotics (ISAPP) provides the most widely accepted definition of postbiotics as “a preparation of inanimate microorganisms and/or their components that confers a health benefit on the host.”

Administration of a powdered formulation containing Ascophyllum nodosum over a 6-week period in dogs and cats resulted in a significant decrease in dental plaque and calculus [20]. Several human studies have shown the benefits of probiotics in reducing the growth of oral pathobionts and improving oral health, for example, a reduction in the growth of *Porphyromonas gingivalis*, *Prevotella intermedia*, and *Fusobacterium nucleatum* in vitro by strains of *Lactobacillus reuteri*, *Streptococcus salivarius*, and *S. oralis*, and a reduction in plaque formation by *Levilactobacillus brevis* and *Lactiplantibacillus plantarum* [21,22,23]. A recent in vitro study demonstrated that the addition of the prebiotic nitrate to salivary-derived biofilms from twelve healthy human participants significantly decreased bacterial genera associated with caries and periodontitis [24]. The extent to which human data can be extrapolated to dogs is unclear; however, given the differences between the oral microbiome of humans and dogs, this should be performed with caution. Indeed, a recent comparison of the microbiome of dental plaque of dogs and humans revealed that there was little overlap between species (5.9% microbial species in common) [25]. With mounting evidence for the use of pro- and postbiotics to induce beneficial changes in the oral microbiome in humans, this highlights the need for further research in this nascent field in dogs.

The aim of this study was to identify a candidate ingredient to support oral health in dogs through the application of probiotics and postbiotics to a screening pipeline of models relevant to canine oral health. The study consisted of both a preclinical and clinical phase. The individual performance of candidate strains was assessed in vitro. Assessments included the candidate’s ability to inhibit the growth and formation of biofilm of canine oral species; modify the growth and composition of oral biofilms originating from saliva samples from twelve dogs using the xCELLigence Real Time Cell Analysis (RTCA) assay; and inhibit inflammatory markers in a buccal epithelial cell model. The clinical phase aimed to use the leading candidate in a clinical trial of 60 dogs to establish its efficacy on canine oral health, and to correlate the microbiome with oral health scores for 1gingivitis, halitosis, plaque, and calculus.

## 2. Methods

### 2.1. Preclinical Phase

#### 2.1.1. Strain Preparation and Characterisation

Probiotic strains (Supplementary Table S10) were cultured in de Man, Rogosa, and Sharpe (MRS) broth (BD, San Jose, CA, USA) under anaerobic conditions at 37 °C for 24 h. Postbiotics were obtained by heat-treating the probiotic strains; probiotic cultures were suspended in 0.9% salt solution before boiling at 100 °C for 1 h. HT cultures were then concentrated and lyophilised without any cryoprotectant or carrier addition. To confirm cell inactivation by heat treatment, live/dead cell staining was performed using 0.1 nM SYTO9 (Thermo Fisher Scientific, Waltham, MA, USA) and 30 nM propidium iodide (Sigma-Aldrich, St. Louis, MO, USA) and cells analysed by flow cytometry (CytoFlex; Beckman Coulter, Brea, CA, USA) (Supplementary Materials). The potency or enumeration of the HT cells was determined by flow cytometry following the same procedure. Further confirmation of cell inactivation by heat treatment was performed through culture of cells on MRS agar plates.

#### 2.1.2. Growth Inhibition of Canine Oral Early Colonizers

Candidate strains were added to brain–heart infusion (BHI) broth with 10^7^ colony forming units (CFU)/mL of the canine oral early colonizer strain suspensions (Supplementary Materials), as previously described [22]. The growth inhibition of canine oral early colonizer strains was tested using different doses of HT *L. plantarum* CECT 9161 (10^6^, 10^7^, 10^8^ and 10^9^ cells/mL). The different treatments were cultured for 24 h at 37 °C, 330 rpm before cultures were spotted onto BHI agar plates and cultured at 37 °C for 24 h to determine CFU counts.

#### 2.1.3. Inhibition of Oral Early Colonizer Biofilm Formation

Three doses of candidate strains (10^7^, 10^8^, and 10^9^ cells/mL) were suspended in BHI medium and 100 µL of each dose was added in triplicate to a multi-well plate with 100 µL of BHI media and the canine oral early colonizer strain suspensions (optical density [OD]_595_ = 0.13). The plates were incubated at 37 °C for 24 h. After incubation, the supernatant was removed, and biofilms were washed three times with 0.9% sodium chloride solution. Biofilm detection was conducted by incubation for 1 h at 60 °C before staining with 150 µL of 1% crystal violet for 15 min at room temperature. After staining, the crystal violet was removed, the wells were washed twice with 150 µL of Elix^®^ water (Merck, Darmstadt, Germany), and the plate was dried at 37 °C for 15 min. Finally, 150 µL of alcohol was added to each well and the plate read at 540 nm using a multi-scan spectrophotometer (Multiskan Ascent; Thermo Fisher Scientific, Waltham, MA, USA), as previously described [26].

#### 2.1.4. Influence of HT *L. Plantarum* CECT 9161 on Growth and Microbial Composition of Biofilms from Canine Saliva

The influence on biofilm development was determined using the xCELLigence RTCA assay (Agilent, Santa Clara, CA, USA), as previously described [27]. Briefly, 50 μL of canine saliva from each dog (*N* = 12) was added to 100 µL of BHI medium with vitamin K1 and hemin, resulting in twelve inocula that each represented an individual dog. Then, 100 µL of BHI with or without postbiotic was added. The postbiotics used included HT *L. plantarum* CECT 9161, and strains designated “HT *Lactiplantibacillus plantarum* 1”, “HT *Lactiplantibacillus reuteri* 1”, and “HT *Lactiplantibacillus reuteri* 2” for the purposes of the study. The final well concentration was 10^9^ cells/g. Two controls were used: BHI without postbiotic and postbiotic without saliva inoculum as an analytical control. The assay was run for 12 h, and the pH was measured at 12 h in all wells. Biofilm formation was studied by impedance with E-plates 96 (Agilent, Santa Clara, CA, USA) [27]. Details of the DNA isolation and amplicon sequencing of biofilm samples can be found in the Supplementary Materials.

#### 2.1.5. Statistical Analysis of In Vitro Microbiome Features

Taxa data were normalised using the rarefaction technique from the Phyloseq R package (R Foundation for Statistical Computing, Vienna, Austria, www.R-project.org (URL accessed on 1 November 2022) v1.34 to perform alpha diversity analysis. Shannon, Simpson, and Richness indices were calculated using the vegan R package v2.5-7 [28], and the Wilcoxon signed-rank test was used to determine significance in alpha diversity between groups. Bray–Curtis dissimilarity matrix and permutational multivariate analysis of variance (PERMANOVA) for beta diversity were performed using the vegan R package v2.5-7 after normalisation by relative frequency for each sample. Characterisation of in vitro microbiota biofilms can be found in the Supplementary Materials.

#### 2.1.6. Modulation of Inflammatory Markers in a Buccal Epithelial Cell Model

The TR146 cell line (abm^®^, Richmond, BC, Canada) derived from a human squamous carcinoma of the buccal mucosa was used in this model because of the lack of a commercially available canine buccal cell line. Cells were grown in Ham’s F12 medium supplemented with 10% foetal bovine serum (FBS; Thermo Fisher Scientific, Waltham, MA, USA), 2 mM L-glutamine, and penicillin–streptomycin (100 U/mL–100 µg/mL) (Thermo Fisher Scientific, Waltham, MA, USA) at 37 °C in 5% CO_2_. TR146 were seeded in 96-well plates (4 × 10^4^ cells/well) and cultured for 7 days to form cell monolayers. On the day of the experiment, the cells were washed once with phosphate-buffered saline (PBS; Thermo Fisher Scientific, Waltham, MA, USA) and then mixtures containing TNF-α (10 ng/mL) and the selected HT strains (10^8^ and 10^9^ cells/mL) were prepared in culture medium without antibiotics. They were immediately added to each well and the plates were incubated for 3 h at 37 °C in 5% CO_2_. After inflammation induction, cell supernatants were harvested and stored at −20 °C until cytokine quantitation. To rule out cytokine inhibition release due to cytotoxicity, cell viability was determined using the 3-(4,5-dimethylthiazol-2-yl)-2,5-diphenyltetrazolium bromide (MTT) assay and data were analysed in GraphPad Prism using a one-way ANOVA with Dunnett’s post hoc test. Cytokine analysis in cell culture supernatants was performed using the Luminex 200™ System with a 3-cytokine/chemokine panel that included IL-6, IL-8 and CXCL10 (Thermo Fisher Scientific, Waltham, MA, USA), according to the manufacturer’s instructions. For each HT strain, the percentage of inhibition for each cytokine was calculated by comparison to positive controls exposed to TNF-α (10 ng/mL) alone.

#### 2.1.7. Statistical Analysis of In Vitro Data

An analysis was conducted in GraphPad Prism 10.2 (GraphPad Software, La Jolla, CA, USA) using the following statistical tests: one-way ANOVA, Dunnett’s post hoc analysis, and Student’s *t*-test. A *p*-value of ≤0.05 was considered statistically significant.

### 2.2. Clinical Phase

#### 2.2.1. Animals

The canine study was conducted at Ontario Nutri Lab Inc, a registered research animal facility in Fergus, ON, Canada. The study was conducted in accordance with OMAFRA, the Animals for Research Act, and the guidelines set forth by the Canadian Council on Animal Care. Ethical approval was obtained from Ontario Nutri Lab’s Internal Animal Care & Use Committee (IACUC) for the clinical study in dogs and the study was performed in accordance with relevant guidelines and regulations. Methods are reported according to ARRIVE guidelines. The dogs were returned to the colony at the end of the clinical trial.

Sixty healthy adult male and female dogs aged ≥1 year were included in the study. Each animal represented an experimental unit. Sample size was calculated using plaque as the primary outcome measure, with a between-group difference of 15%, an estimated control group mean of 4.9, and standard deviation of 0.8 based on in-house data. Changes in gingivitis, halitosis, and calculus scores were secondary outcome measures. The dogs were stratified based on the rate of plaque accumulation assessed in the pretest phase (Supplementary Materials) and were assigned to one of three groups: CON (*n* = 20), LOW (*n* = 20), or HIGH (*n* = 20). The dogs received either a control maltodextrin placebo powder (CON), a low dose of HT *L. plantarum* CECT 9161 (5 mg of 1 × 10^11^ cells/g postbiotic) (LOW), or a high dose of HT *L. plantarum* CECT 9161 (25 mg of 1 × 10^11^ cells/g postbiotic) (HIGH) once daily, formulated into identical capsules. All capsules were indistinguishable in appearance, colour, size, shape, and smell and provided in coded bulk capsule pots. The capsules were opened, and the contents sprinkled on kibble. The kennel staff were blinded to the group allocation. Information on animal husbandry during the study can be found in the Supplementary Materials.

#### 2.2.2. Assessment of Consumption

The consumption of kibble (Purina Dog Chow) was recorded daily. The weight of the diet offered (g) and the diet consumed (g) was recorded for each dog on each day of the study to calculate the percentage of diet consumed each day. The intervention was a small amount (mg) mixed in with the main diet. Since the amount of treatment consumed could not be measured directly, the amount of diet consumed overall was used as a proxy.

To investigate the consumption time of individual dogs to account for differences in the speed of eating and, therefore, the contact time of the postbiotic, the time taken to consume each meal over a three-day period was measured for each dog.

#### 2.2.3. Dental Examinations

The removal of dental deposits by dental scale and polishing was performed under general anaesthesia (Supplementary Materials) on Day 1. An oral examination of each dog was performed on Days 1, 29, and 57. Gingivitis and halitosis were scored under general anaesthesia on Day 1, and the gingivitis, halitosis, plaque, and calculus indices were scored under general anaesthesia on Days 29 and 57. The buccal surfaces of the following teeth on both sides of the mouth were reviewed in the upper and lower jaws: incisor 3, canine, premolar 3, premolar 4, and molar 1. The scorer was blinded to group assignment during the entire trial period. The same staff member scored a particular index (plaque or calculus) for all dogs at each examination. Gingival inflammation was evaluated using the Loe and Silness gingival index [29] and plaque was evaluated using a modified Logan and Boyce plaque index method [30]. A 2% eosin disclosing solution was applied to the teeth and air dried before calculus was scored using the modified Warrick and Gorrel method [31]. Plaque was not removed for calculus scoring. Halitosis was evaluated using a Halimeter, which measures volatile sulphur compounds. Three readings were taken per dog, per timepoint, to calculate an average value.

#### 2.2.4. Supragingival Plaque Sample Collection

Supragingival plaque samples were collected on Day 57 using collection kits from DNA Genotek (Ottawa, ON, Canada). Samples were stored according to manufacturer’s instructions at room temperature until analysis.

#### 2.2.5. Statistical Analysis of Gingivitis, Halitosis, Plaque, and Calculus Scores

A linear model was fitted to each of the four outcome variables: gingivitis, halitosis, plaque, and calculus. Scores for gingivitis and halitosis were calculated as change from baseline to Day 29 and baseline to Day 57. Plaque and calculus scores at Days 29 and 57 were considered equivalent to the change from baseline due to teeth cleaning at Day 0. A mixed-effects linear model, including a random effect for dogs, was used with fixed-effects including intervention (CON, LOW, or HIGH), time (change at Day 29 and Day 57), and baseline measures for gingivitis and halitosis (tartar and plaque baseline values were 0 for all dogs due to teeth cleaning). Time to consume diet (minutes), mean diet consumed (%), and an interaction between the time and intervention fixed-effects were also explored as fixed effects, but only included in the final model if they were found to be statistically significant (based on an *F*-test). Reported model fit was checked by using plots of the residuals for all models. Analysis was undertaken using R version 4.3.1, package lme4 and lmerTest for the mixed-effects models [32,33], and testing and ggeffects to calculate marginal means (predicted changes) [34].

#### 2.2.6. DNA Isolation and Metagenomic Next-Generation Sequencing of Supragingival Plaque Samples

The microbiome DNA extraction of supragingival plaque samples included enzymatic lysis using 100 mg/mL lysozyme, 1 mg/mL lysostaphin, and 25 KU/mL mutanolysin (Sigma-Aldrich, St. Louis, MO, USA) for 30 min at 37 °C; bead beating: FastPrep-24-5G, one round of 60 s at 6.0, followed by processing of samples using the QIAsymphony PowerFecal Pro DNA Kit (Qiagen, Barcelona, Spain) robotic magnetic bead-based kit. DNA concentration was assayed using the Qubit dsDNA System (Thermo Fisher Scientific, Waltham, MA, USA) and samples were normalised accordingly to generate high-quality functional libraries.

#### 2.2.7. Next-Generation Sequencing of Supragingival Plaque Samples

Libraries were prepared with the Nextera XT Library kit (Illumina San Diego, CA, USA) following the manufacturer’s instructions. Library quality control was ensured by profiling and length distribution analysis using an HSD5000 kit in the TapeStation 4200 equipment (Agilent, Santa Clara, CA, USA). The libraries were sequenced on the NovaSeq 6000 platform in 150 paired-end reads attaining a minimum of 20 million reads per sample. The configuration generated *.bcl files as primary sequencing output (NovaSeq Control Software (NCS) v.1.6). The Bcl2fastq v.2.20 program was used to translate the sequencing reads from bcl (Base Calling) to FASTQ format. This step also removed sequencing adapters (data deposited in NCBI SRA under BioProject PRJNA1150971).

#### 2.2.8. Preprocessing for Metagenome Analysis

The Clumpify tool from the BBToolssuite [35] was used to remove optical duplicates. Reads with a phred quality score less than Q20 and length less than 50 nts were filtered out using the program BBMap v38.36. Human and dog genome presence was filtered using NGLess (v.1.0.0) [36] using the built-in Homo Sapiens “hg19” genome as a first reference and “GCF_000002285_CanFam3.1_genomic” genome as a second reference. Those reads with alignments with more than 45 bases and 97% similarity to the reference genome were discarded. The remaining sequences were called “high-quality sequences” and were intended to be the final sequences.

Metaphlan (v.4) was used to assign the taxonomy [37]. The reads of each sample were aligned against the “CHOCOPhlAn” database that contains single-copy genetic markers present in almost all bacteria, specifically the January 2021 version (mpa_vJan21_CHOCOPhlAnSGB_202103) for the preclinical phase and the October 2022 version (mpa_vOct22_CHOCOPhlAnSGB_202212) for the clinical phase. From these alignments, the estimated number of reads contributed to a given clade for each identified taxon were obtained computationally.

MEGAHIT genome assembler (v.1.2.9) was used to perform the assembly of the “high-quality sequences” [38]. Six assemblies were conducted at k-mer sizes of 21, 33, 55, 77, 99, and 127 and the automatically selected “final.contigs.fa” file was used, which corresponded to the 127 k-mer size assembly for both preclinical and clinical phase cases. Contigs larger than 500 bp were used to predict genes using Prodigal (v.2.6.3) [39,40]. KEGG functional annotation was performed on the predicted genes using the web server GhostKoala [41]. The gene quantification was calculated with SALMON software (v.1.5.1) [42]. Reads from genes from heme biosynthesis module were aligned using BLASTn [43] against nt database (version July 2022) to study the taxonomy of those genes.

#### 2.2.9. Statistical Analysis of the Supragingival Plaque Microbiome

The relative taxonomic abundances of the samples were displayed with collapsed histograms plotted by “ggplot2” (v.3.4.0) library in R version 4.2.3 [44]. Taxa and genes were normalised using the rarefaction technique from “phyloseq” (v.1.34) R package [45] to perform alpha diversity analysis. Shannon, Simpson, and Richness indices were calculated using the “vegan” (v.2.5-7) R package [46]. Violin plots were created with the “ggpubr” (v.0.4.0) library in R [47], and a Wilcoxon signed-rank test was performed through the “stats” (v.3.6.0) R package to determine significance between groups. To illustrate taxonomic dissimilarities based on amplicon sequence variants (ASVs), Principal Coordinate Analysis (PCoA) across the samples was carried out using the Bray–Curtis distance matrix calculated with the “phyloseq” (v.1.034) R package [45] and represented with “ggplot” (v.3.4.0) R package [44]. The effects of the factors on taxonomy data were evaluated with PERMANOVA with the “vegan” (v.2.5-7) R package [46] using the Bray–Curtis dissimilarity matrix that was previously calculated considering the relative abundances of functional categories in all samples.

#### 2.2.10. Identification of Taxonomic and Functional Features in the Microbiome of Supragingival Plaque

Differential taxa and gene abundance analysis was performed using the DESeq2 R package v.1.30.1 [48]. The normalisation was based on the “Relative Log Expression” method. The “EstimateSizeFactors” function was used to calculate the scaling factors using the median ratio between taxon and gene abundances and the geometric mean. The “poscounts” method was used to address the taxa and genes that had multiple zeros in the samples. A taxon was considered differentially abundant with a Benjamini–Hochberg (“BH”) multiple testing correction adjusted *p* < 0.05 and if it was present in ≥50% of the samples of one of the compared groups. To determine significance of the functional profiles between groups, a Gene Set Enrichment Analysis (GSEA) was additionally conducted in the metagenomic dataset using fgsea (v.1.16) R package [46] on KEGG modules. The “stat” statistic of genes from DESeq2 differential abundance analysis was used to rank the genes to perform the GSEA. Heatmaps were constructed using the ComplexHeatmap R package v.2.11.1 [49].

## 3. Results

A library of microorganisms and microbe-derived ingredients, including potential probiotics isolated from healthy humans and dogs, were screened in the preclinical phase of the present study. All biotic ingredients were investigated for their ability to inhibit the growth and biofilm formation of canine oral biofilm early colonizers: *N. zoodegmatis*, *N. weaveri*, and *Pasteurella dagmatis* [11]. The most inhibitory probiotics were tested in their postbiotic form in a canine-saliva-derived biofilm model using the xCELLigence RTCA assay and a buccal epithelial cell model.

### 3.1. Growth Inhibition of Canine Oral Biofilm Early Colonizers

**Figure 1:**
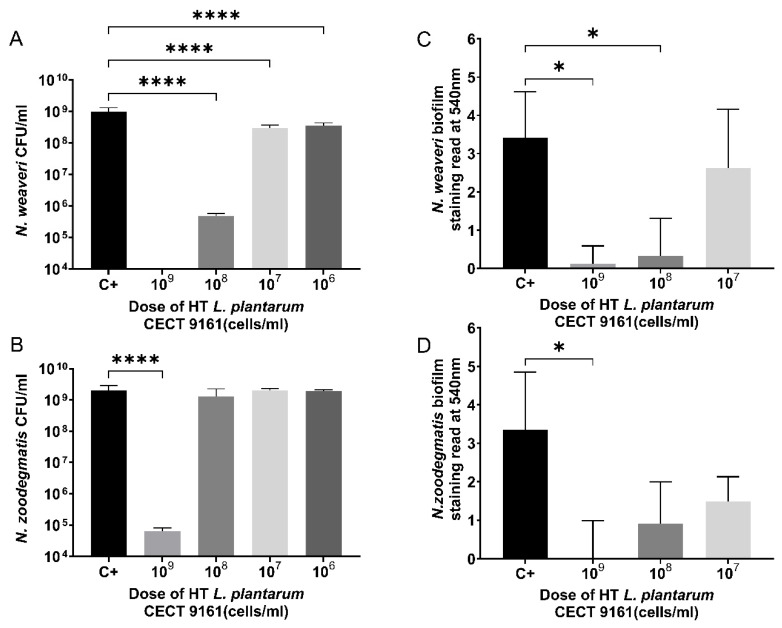
Inhibitory effect of HT *L. plantarum* CECT 9161 on the growth of canine oral early colonizers in vitro. (**A**) Growth inhibition of *N. weaveri* when co-cultured with HT *L. plantarum* CECT 9161. Complete growth inhibition was observed at a dose of 10^9^ cells/mL. (**B**) Growth inhibition of *N. zoodegmatis* when co-cultured with HT *L. plantarum* CECT 9161. Significant growth inhibition occurred at a dose of 10^9^ cells/mL. (**C**) Effect of HT *L. plantarum* CECT 9161 on growth of *N. weaveri* biofilm. Greatest inhibition observed at the highest dose (10^9^ cells/mL). (**D**) Effect of HT *L. plantarum* CECT 9161 on growth of *N. zoodegmatis* biofilm. Greatest inhibition observed at the highest dose (10^9^ cells/mL). Data are represented as means ± SD of three technical replicates. Statistical significance was tested in GraphPad Prism using a one-way ANOVA with post hoc Dunnett’s test. * *p* < 0.05, **** *p* < 0.0001. C+, control; CFU, colony forming unit; HT *L. plantarum* CECT 9161, heat-treated *Lactiplantibacillus plantarum* CECT 9161; *N. zoodegmatis*; *Neisseria zoodegmatis*; *N. weaveri*; *Neisseria weaveri*; SD, standard deviation.

Heat-treated (HT) *Lactiplantibacillus plantarum* CECT 9161 was associated with the inhibition of canine oral early colonizers, with evidence of inhibition in a dose-dependent manner. The complete inhibition of *N. weaveri* growth (Figure 1A) and significant growth inhibition of *N. zoodegmatis* (*p* < 0.0001) (Figure 1B) were observed when cultured in the presence of HT *L. plantarum* CECT 9161 at a dose of 10^9^ cells/mL. In biofilm experiments, the greatest inhibition of *N. weaveri* (*p* < 0.05) and *N. zoodegmatis* (*p* < 0.05) single-species biofilms was observed at the highest dose (10^9^ cells/mL) of HT *L. plantarum* CECT 9161 (Figure 1C,D). The significant inhibition of *P. dagmatis* ATCC 51570 biofilms was also observed in a dose-dependent manner: 10^9^ cells/mL (*p* < 0.0001), 10^8^ cells/mL (*p* < 0.001), and 10^7^ cells/mL (*p* < 0.05) (Supplementary Figure S1A).

### 3.2. Influence of HT L. plantarum CECT 9161 on Growth and Microbial Composition of Biofilms from Canine Saliva

**Figure 2:**
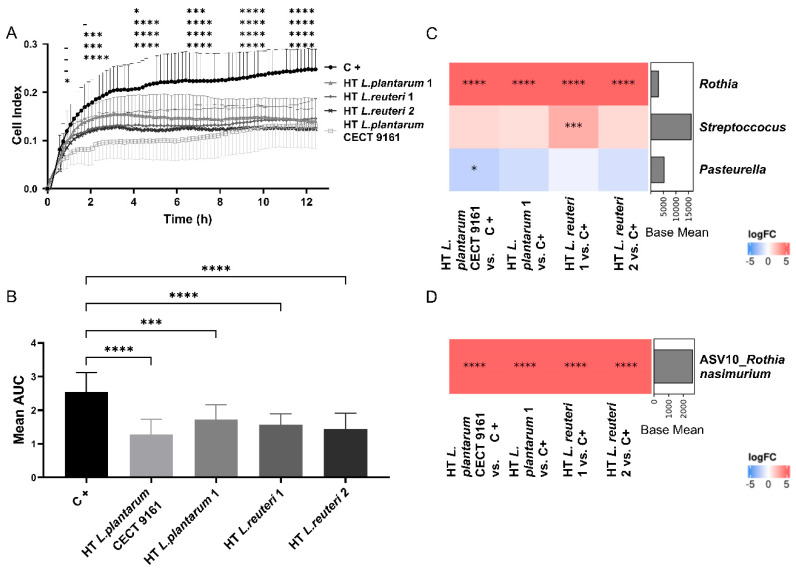
Influence of HT *L. plantarum* CECT 9161 on growth and microbial composition of biofilms from canine saliva. (**A**) Quantification of biofilm formation over a 12 h incubation period from canine saliva samples (*N* = 12) determined by impedance data from the xCELLigence RTCA assay. HT *L. plantarum* CECT 9161 demonstrated a significant decrease in the Cell Index curve. Mean (± SD for 12 replicates) represented at each timepoint. (**B**) AUC analysis of xCELLigence RTCA assay data on biofilm growth presented in (**A**). HT *L. plantarum* CECT 9161 shows the greatest reduction in biofilm growth. Statistical significance was tested in GraphPad Prism using a one-way ANOVA with Dunnett’s post hoc analysis. * *p* < 0.05, *** *p* < 0.001, **** *p* < 0.0001. LogFC of deferentially abundant bacterial taxa of the DNA obtained from biofilms grown (**A**) at genus (**C**) and ASV level (**D**). Red on the heatmap indicates that the bacteria had a higher abundance in the postbiotic than the control (C+) and blue indicates a lower abundance. Statistical analysis was tested in R with the “limma-voom” package using its internal moderated *t*-test. * *p* < 0.05, *** *p* < 0.001, **** *p* < 0.0001. ASV, amplicon sequence variants; AUC, area under the curve; C+, control; DNA, deoxyribonucleic acid; h, hours; HT *L. plantarum* CECT 9161, heat-treated *Lactiplantibacillus plantarum* CECT 9161; HT *L. plantarum* 1, heat-treated *Lactiplantibacillus plantarum* 1; HT *L. reuteri* 1, heat-treated *Lactiplantibacillus reuteri* 1; HT *L. reuteri* 2, heat-treated *Lactiplantibacillus reuteri* 2; logFC, log fold change; RTCA, Real-Time Cell Analysis; SD, standard deviation.

HT *L. plantarum* CECT 9161, and strains designated “HT *Lactiplantibacillus plantarum* 1”, “HT *Lactiplantibacillus reuteri* 1”, and “HT *Lactiplantibacillus reuteri* 2” for the purposes of the study, were incubated with saliva to form in vitro biofilms during a period of 12 h. The addition of HT *L. plantarum* CECT 9161 resulted in a significant decrease (*p* < 0.001) in the Cell Index curve, indicating decreased biofilm formation and development by salivary microorganisms (Figure 2A). Biofilm growth was investigated by calculating the area under the curve of impedance data for each experiment. HT *L. plantarum* CECT 9161 was associated with the greatest reduction in biofilm growth (*p* < 0.0001) (Figure 2B). pH was not affected by HT *L. plantarum* CECT 9161 (Supplementary Figure S1C).

A 16s rRNA analysis of the in vitro biofilms from the saliva of 12 individual dogs showed complex multispecies biofilm formation with the modulation of microbial composition resulting from growth in the presence of HT *L. plantarum* CECT 9161. Presumptive species designations based on nearest known matches identified from NCBI 16s rRNA databases showed that HT *L. plantarum* CECT 9161 was associated with an increased abundance of nitrate-reducing bacteria belonging to the family *Micrococcaceae* (adjusted [adj.] *p* < 0.0001) (Supplementary Figure S1B), genus *Rothia* (adj. *p* < 0.0001) (Figure 2C), and species *Rothia nasimurium* (ASV10; adj. *p* < 0.0001) (Figure 2D) compared with the control. A significant decrease in the abundance of *Neisseriaceae* (adj. *p* = 0.0334) (Supplementary Figure S1B) and *Pasteurella* (adj. *p* = 0.0141) (Figure 2C) was also observed in biofilms grown with HT *L. plantarum* CECT 9161 compared with the controls. Both taxonomical groups included canine oral early colonizers [10].

### 3.3. Modulation of Inflammatory Markers in a Buccal Epithelial Cell Model

**Figure 3:**
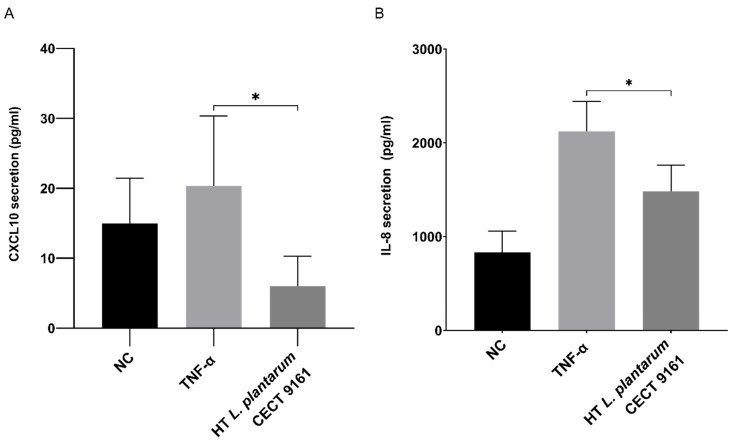
Effect of HT *L. plantarum* CECT 9161 on inflammatory markers in a buccal epithelial cell model. TR146 cells were co-cultured with HT *L. plantarum* CECT 9161 at 37 °C in 5% CO_2_. Cell supernatants were harvested after the induction of inflammation for cytokine quantification, which was performed using the Luminex 200™ System. HT *L. plantarum* CECT 9161 significantly inhibited IL-8 (*p* = 0.0362) (**A**) and CXCL10 (*p* = 0.0107) (**B**) compared to the TNF-α positive control. Data are represented as means ± SEM of two independent experiments with three technical replicates. Statistical significance was tested in GraphPad Prism using a one-way ANOVA with Dunnett’s post hoc analysis. * *p* < 0.05. CO_2_, carbon dioxide; CXCL10, C-X-C motif chemokine ligand 10; HT *L. plantarum* CECT 9161, heat-treated *Lactiplantibacillus plantarum* CECT 9161; IL-8, interleukin 8; NC, negative control; TNF-α, tumour necrosis factor alpha; SEM, standard error of the mean.

Cells derived from human buccal mucosa were incubated with TNF-α and HT *L. plantarum* CECT 9161 to investigate the effect of the postbiotic on the production of pro-inflammatory cytokines. This assay revealed the suppression of pro-inflammatory cytokine production by the postbiotic. HT *L. plantarum* CECT 9161 significantly inhibited the secretion of IL-8 (29 ± 3% of control; *p* = 0.0362) and CXCL10 (72 ± 6% of control; *p* = 0.0107) compared with the TNF-α positive control (Figure 3). No cytotoxicity was observed (*p* = 0.36).

### 3.4. Clinical Phase

Sixty dogs were stratified based on the rate of plaque accumulation assessed in a pretest phase and were assigned to one of three groups: CON (*n* = 20), LOW (*n* = 20), or HIGH (*n* = 20). The dogs received either a control maltodextrin placebo powder (CON), a low dose of HT *L. plantarum* CECT 9161 (5 mg of 1 × 10^11^ cells/g postbiotic) (LOW), or a high dose of HT *L. plantarum* CECT 9161 (25 mg of 1 × 10^11^ cells/g postbiotic) (HIGH) once daily. Data were analysed from all 60 dogs included in the study over the 57-day trial period. Typically, most dogs consumed a high proportion of their diet on each day (close to 100%) and consumed their diet within five minutes.

### 3.5. Oral Health Markers

**Figure 4:**
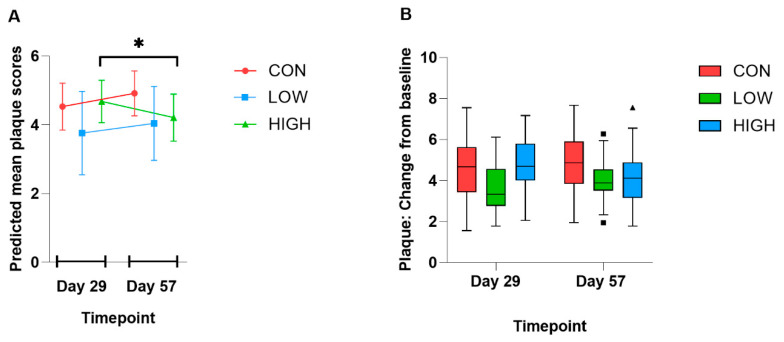
Change in plaque from baseline at Days 29 and 57. Dogs (*N* = 60) were stratified into three intervention groups (CON [*n* = 20], LOW [*n* = 20], or HIGH [*n* = 20]) and underwent tooth cleaning on Day 0. Plaque scores were measured at Days 29 and 57 for each dog in the study. (**A**) Graph showing predicted mean (with 95% CI as error bars) plaque scores in each intervention group at Days 29 and 57 estimated from the fixed-effects model. Plaque scores in the HIGH group significantly decreased at Day 57 compared with Day 29 (estimated effect −0.86; 95% CI: −1.61, −0.11; *p* = 0.031). There was a tendency for lower plaque accumulation in the LOW group compared with the CON group (estimated effect −0.77; 95% CI: −1.58, 0.04; *p* = 0.07). (**B**) Boxplots showing the change in plaque scores from baseline in each intervention group at Days 29 and 57. The box includes the upper and lower quartiles and, therefore, the middle 50% of the data and the horizonal line within the box are the median. The whiskers extend to 1.5 times the interquartile range and data points outside of this range are marked as squares (LOW) or triangles (HIGH). A mixed-effects linear model with fixed-effects baseline plaque scores, time, intervention, and time interaction, as well as dog random effect and subsequent F-test, was used for analysis and *p* < 0.05 was deemed statistically significant. * *p* < 0.05. CI, confidence interval; CON, control group; HIGH, high-dose heat-treated *Lactiplantibacillus plantarum* CECT 9161 group; LOW, low-dose heat-treated *Lactiplantibacillus plantarum* CECT 9161 group; *N*, total number of dogs; *n*, number of dogs in each intervention group.

The three intervention groups were equivalent at baseline for gingivitis and halitosis scores (Supplementary Table S1). There was no overall intervention effect detected for the oral health markers: gingivitis, halitosis, and calculus (Supplementary Tables S2–S9). There was a non-significant tendency for an intervention*time effect on plaque scores, with lower plaque accumulation in the LOW group compared with the CON group (estimated effect −0.77; 95% confidence interval [CI]: −1.58, 0.04; *p* = 0.07). Plaque scores in the HIGH group significantly decreased at Day 57 compared with Day 29 (estimated effect −0.86; 95% CI: −1.61, −0.11; *p* = 0.03 [Figure 4 and Supplementary Tables S8 and S9]). There was a non-significant reduction in calculus formation in the LOW group compared with CON (estimated effect −0.40; 95% CI: −0.87, 0.07; *p* = 0.10).

General trends were also observed: an increase in halitosis at Day 29 compared with baseline for all groups and a significant decrease in halitosis at Day 57 compared with Day 29; calculus was significantly increased in all groups at Day 57 compared with Day 29 (Supplementary Figure S2).

### 3.6. Supragingival Plaque Samples: Microbiota Diversity and Composition

The bacterial composition of supragingival plaque was determined and taxonomic classification was assigned to between 28% and 50% of sequences from all samples (*N* = 60). Within the classified species, 153 distinct species were bacteria and archaea, of which species of the *Porphyromonas* genus dominated (*Porphyromonas gulae* [14.54 ± 5.49%], *Porphyromonas cangingivalis* [11.78 ± 4.24%], and *Porphyromonas canoris* [10.31 ± 4.42%]). Shannon alpha diversity was significantly higher in the HIGH group compared with the LOW group; no differences in Richness or Simpson indices were observed (Supplementary Figure S3A). Differentially abundant genera were not observed between groups. No clustering by intervention was observed using PCoA of the Bray–Curtis distance constructed at species level (Supplementary Figure S3B).

**Figure 5:**
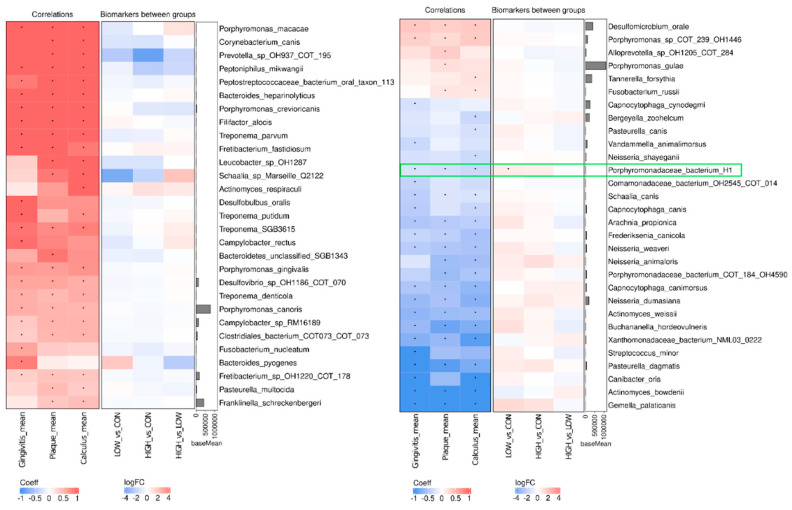
Differential abundance analysis of the microbial composition of supragingival plaque. (**Left**): Heatmap of the correlation coefficients between clinical markers and species abundance. (**Right**): Heatmap of the logFC of group comparisons at the species level. Red shading indicates a direct correlation (**left panel**) or increased abundance in the first group of the comparison compared with the second (**right panel**); blue shading indicates inverse correlation (**left panel**) or increased abundance in the second group of the comparison compared with the first (**right panel**). On the right side, the significance takes into consideration that the taxon must be present in a minimum of 50% of the samples of ≥1 of the two compared groups. The bar plot shows the mean normalised abundance of each taxon. Clades without a valid name were not represented. Several species were associated with gingivitis, plaque, and calculus scores. *Porphyromonadaceae* bacterium H1 was significantly increased in the LOW group when compared with the CON group. Statistical significance of correlations was tested in R with the “Maaslin2” package using its internal test, and for group comparisons it was tested with “the DESeq2” package using its internal Wald test. * adj. *p* < 0.05. adj., adjusted; CON, control group; HIGH, high-dose heat-treated *Lactiplantibacillus plantarum* CECT 9161 group; LOW, low-dose heat-treated *Lactiplantibacillus plantarum* CECT 9161 group; logFC, log fold change.

Several species were positively correlated with gingivitis, plaque, and calculus scores, including *Porphyromonas macacae*, *Corynebactum canis*, *Peptoniphilus mikwangi*, *Porphyromonas crevoricanis*, *Filofactor alocis*, *Treponema parvum*, *Fretibacterium fastidiosum*, and species of *Prevotella* and *Peptostreptococcaceae*, indicating a negative association with oral health (Figure 5). The abundance of other species, including *Gemella palaticanis*, *Actinomyces bowdenii*, *Canibacter oris*, *P. dagmatis* ATCC 51570, and species of *Neisseria*, were negatively correlated with gingivitis, plaque, and calculus scores, indicating a positive association with oral health. The abundance of *Porphyromonadaceae bacterium* H1 significantly increased (log2FC = 0.721 ± 0.220, adj. *p* = 0.037) in the LOW group when compared with the CON group and was inversely correlated with gingivitis, plaque, and calculus scores, indicating a positive association with oral health (Figure 5).

### 3.7. Supragingival Plaque Samples: Microbiota Functional Gene Composition

**Figure 6:**
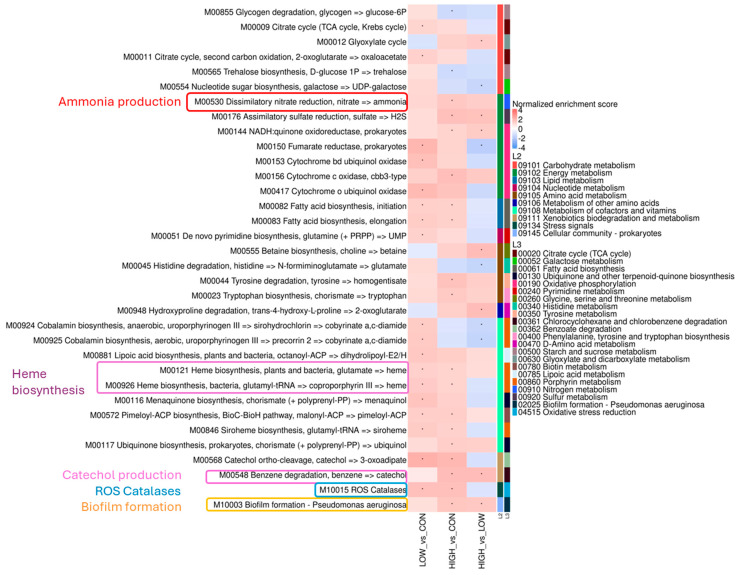
GSEA of KEGG modules for supragingival plaque microbial composition. Heatmap of the NES of KEGG module abundances. Columns show the results of comparisons between groups. Red shading indicates positive NES values, meaning the module is enriched in the first group of the comparison. Blue shading indicates negative NES values, meaning the module is enriched in the second group of the comparison. Legend “L2” refers to KEGG annotation at general category level, while “L3” refers to KEGG annotation at pathway level. Only complete modules are depicted. Several genes were differentially expressed that could be associated with oral health, including H_2_S and ammonia production, tryptophan and heme biosynthesis, catechol production, ROS catalases, and biofilm formation. Statistical significance was tested in R with the “fgsea” package by the FDR. * adj. *p* < 0.05. adj., adjusted; CON, control group; FDR, false discovery rate; GSEA, gene set enrichment analysis; H_2_S, hydrogen sulphide; HIGH, high-dose heat-treated *Lactiplantibacillus plantarum* CECT 9161 group; LOW, low-dose heat-treated *Lactiplantibacillus plantarum* CECT 9161 group; NES, Normalised Enrichment Score; ROS, reactive oxygen species.

A GSEA was conducted to compare the differential module enrichment between groups based on the abundance of genes associated with the KEGG database (Figure 6). The abundance of genes involved in hydrogen sulphide (H_2_S; Normalised Enrichment Score [NES] = 1.769, adj. *p* = 0.002) and ammonia production (NES = 1.548, adj. *p* = 0.015) increased in the HIGH group compared with the CON group. Abundance of genes involved in tryptophan biosynthesis (NES = 1.520, adj. *p* = 0.001) and catechol production (NES = 1.840, adj. *p* = 0.017) was increased in the HIGH group compared with the CON group. For the LOW and HIGH groups, the abundance of genes involved in reactive oxygen species (ROS) catalases (NES = 1.790, adj. *p* = 0.003; NES = 1.768, adj. *p* = 0.003, respectively) were increased compared with the CON group. The abundance of genes involved in heme biosynthesis was also increased for the LOW and HIGH groups compared with the CON group (NES = 1.306, adj. *p* = 0.007; NES = 1.224, adj. *p* = 0.024, respectively), and a BLASTn alignment of the genes against the NCBI nt database (July 2022 version) indicated that, of those genes that had a match, most were taxonomically classified as *Porphyromonas cangingivalis* (33.98 ± 9.58%) and *Porphyromonas gingivalis* (17.38 ± 6.65%) (Supplementary Figure S4). Finally, the abundance of genes involved in biofilm formation were increased in the HIGH group compared with both the LOW and CON groups (NES = 1.590, adj. *p* = 0.001 and NES = 1.539, adj. *p* = 0.002, respectively) (Figure 6). A detailed analysis of modules with significant gene enrichment results in between-group comparisons revealed that module M00529 (denitrification) was significantly enriched in the HIGH group compared with the CON group. However, this module is incomplete, as no mapped reads were found against subunit C of the *nor* gene (Supplementary Figure S5).

## 4. Discussion

Both the quantity and microbial composition of the canine plaque biofilm are interconnected with oral health [9,14,15]. Presently, most strategies to improve oral health in dogs focus on mechanical plaque removal only, and the opportunity to influence microbial composition and function is incompletely understood. Recent evidence has shown that some microorganisms can exert health benefits in their inactivated (postbiotic) form [50]. Postbiotics enable incorporation into delivery formats that impair microbial viability, such as dental chews and kibble. This area of biotics research—postbiotics for canine oral health—can best be described as nascent, with few relevant published interventional trials to date. This study describes the identification, characterisation, and testing in vivo of the novel canine oral health postbiotic HT *L. plantarum* CECT 9161. The investigation revealed that *L. plantarum* CECT 9161 modulated the metagenome of supragingival plaque to reduce dental plaque formation.

Maturation of the plaque biofilm is associated with the development of anaerobic and microaerophilic niches that favour periopathogens [10,51]. Theoretically, the inhibition of tooth colonization may reduce or delay plaque accumulation. In the first phase of the preclinical study, HT *L. plantarum* CECT 9161 was selected for further investigation, having demonstrated in vitro a pronounced ability to inhibit the growth of, and biofilm formation by, the dominant canine oral species *N. weaveri*, *N. zoodegmatis*, and *P. dagmatis*. In the second phase of the preclinical study, canine saliva was used as the inoculum in a novel in vitro assay to investigate the potential for candidate postbiotics to influence the growth and composition of canine oral biofilms. To the authors’ knowledge, this is the first study to use the xCELLigence RTCA assay to investigate the modulation of canine oral biofilm. HT *L. plantarum* CECT 9161 was observed to inhibit the growth and composition of canine-saliva-inoculated oral biofilms. Microbiome analysis of biofilms obtained from the xCELLigence RTCA assay revealed an association between HT *L. plantarum* CECT 9161 and a reduction in the abundance of canine oral early colonizers belonging to the *Neisseriaceae* family and the *Pasteurella* genus. These findings support the assumption that the inhibition of these early colonizers may, in turn, lead to reduced biofilm growth and development. Unexpectedly, HT *L. plantarum* CECT 9161 was associated with an increased abundance of nitrate-reducing *Rothia* species. In the final phase of the preclinical study, HT *L. plantarum* CECT 9161 demonstrated the ability to reduce the secretion of the proinflammatory cytokines IL-8 and CXCL-10, indicating a possible anti-inflammatory effect in the oral cavity.

In the canine clinical study, the oral administration of HT *L. plantarum* CECT 9161 as a top-dress to a standard dry kibble diet was associated with a non-significant tendency towards a reduction in plaque formation of 17% compared to the control in the LOW group, and a significant reduction in plaque of 10% between the midpoint and end of the study in the HIGH group. A threshold for clinical significance of a 15% reduction in plaque accumulation between the intervention and control groups is typically cited as meaningful in dogs by the Veterinary Oral Health Council (VOHC). Considering that the high-dose postbiotic was associated with an absolute reduction in plaque as the study progressed, it is possible that administration over a longer intervention period would achieve the VOHC definition of clinical relevance. Although the threshold for clinical relevance was met in the low-dose group, statistical significance was not (*p* = 0.07). Considering the subjective nature of plaque scoring and the variability in this metric observed in the present study, it is possible that underpowering was responsible for the lack of statistical significance observed. The number of available dogs constrained the sample size used in the clinical trial, therefore further work investigating the low-dose postbiotic in a two-arm trial would enable sample size and statistical power to be increased. No association between HT *L. plantarum* CECT 9161 and calculus formation or gingivitis was observed, despite these being sequelae of plaque formation. It is possible that the study was underpowered to detect a change in these measures, or that the duration was insufficient for an intervention effect to develop. The short intervention period may also have accounted for the comparatively modest reduction in plaque formation, which may become more evident over time as plaque accumulation is expected to increase.

Modulation of the taxonomic composition of the oral microbiome by the postbiotic was observed; specifically, HT *L. plantarum* CECT 9161 was associated with a significant increase in the abundance of *Porphyromonas* bacterium H1 in the LOW group compared with the CON group. In the present study, *P.* bacterium H1 was also positively associated with plaque, calculus, and gingivitis scores. Other members of the *Porphyromonas* genus were found to have a negative association with oral health scores, supporting previous associations between this genus and periodontitis; however, the abundance of these species was not affected by postbiotic consumption. Several species of the genus *Porphyromonas* (e.g., *P. gulae*) are well-known periodontal pathogens [52,53,54]. They persist and grow in the oral cavity by sequestering heme from their host by invading and, in turn, damaging the epithelial layer, activating the host inflammatory response associated with periodontal tissue destruction [52,53,54]. In the functional analysis of the plaque microbiome, genes related to heme biosynthesis were more abundant in dogs receiving the postbiotic (HIGH and LOW) compared with CON, suggesting that organisms associated with HT *L. plantarum* CECT 9161 consumption have the genetic potential for heme biosynthesis. Genes that had a matching sequence in the nt database were primarily taxonomically classified as *Porphyromonas*. It has previously been reported that species of *Porphyromonas* capable of synthesising their own heme are associated with good canine oral health [10,55]. Davis et al. postulated that *Porphyromonas* species possessing heme biosynthesis pathways could occupy the same ecological niche as pathogenic variants of *Porphyromonas,* with the autonomous capability for heme production imparting metabolic flexibility and a competitive advantage to outcompete periodontal pathogens [51]. It is possible that the competition theory could explain the positive association between *P.* bacterium H1 and oral health scores. This represents an area for future study to better understand the microbiome modulation potential of HT *L. plantarum* CECT 9161.

Interestingly, species representing early colonizers in canine dental plaque, *Neisseria* and *Pasteurella*, were not significantly altered in the samples obtained during this clinical trial, and their abundance was positively associated with oral health scores. Increased abundance of these species may reflect a comparatively less mature and more aerobic biofilm that is unfavourable for pathogenic anaerobic and microaerophilic species. Higher proportions of aerobes in canine dental plaque have previously been associated with the healthy state in studies of canine periodontal disease [51]. In the absence of intervention, microbial communities in humans recover quickly following plaque removal [56]. It is possible that HT *L. plantarum* CECT 9161 inhibited the growth of early colonizers in the early recolonization period, but microbial composition had remodelled to a more mature state by the time of the first supragingival plaque sampling at Day 29. It is also possible that the intervention was did not affect the abundance of early colonizers in the complex oral environment. Further work to investigate the effect of HT *L. plantarum* CECT 9161 on the re-establishment of the oral biofilm shortly after plaque removal may elucidate the mechanism of action.

Several other canine oral commensal bacteria were found in this study to correlate with clinical markers of oral health. Periopathogenic species such as *Treponema denticola, Treponema parvum, Fusarium nucleatum*, and *Porphyromonas gingivalis* were directly correlated with gingivitis, plaque, and calculus scores, supporting an association between these species and negative oral health in dogs. Other species found to correlate with oral health scores have a less well-documented relationship in dogs, including *P. macacae, C. canis,* and *P. mikwangii* (negative health association) and *G. palaticanis, A. bowdenii*, and *C. oris* (positive health association).

An association was found between the high dose of HT *L. plantarum* CECT9161 and an increased abundance of genes associated with the denitrification of nitrate to nitric oxide, and the reduction of nitrate to ammonia (which can be spontaneously converted to oral-health-associated ammonium, depending on the pH of the oral environment [57]). Nitrate and nitrate-reducing species have been investigated for a potential beneficial effect on both oral and general health [56,57,58,59]. Nitrates are proposed to exert their oral effects through inhibition of periopathogen growth and through an anti-inflammatory effect on the host gingiva by nitric oxide, the final product of nitrate reduction [56,57]. Further work to quantify the relationship between HT *L. plantarum* CECT9161 and nitrogen metabolites in saliva would confirm whether the increased functional capacity for denitrification associated with HT *L. plantarum* CECT9161 translates to a physiological effect [60]. Other associations were found between HT *L. plantarum* CECT9161 and microbiome functional potential that could be related to an oral health effect. There was an increase in the abundance of genes involved in catechol production associated with HT *L. plantarum* CECT9161, which has been shown to inhibit oral pathogens [61]. The abundance of genes associated with ROS catalases, which are involved in the reduction of oxidative stress and inflammation [62], were also increased in dogs administered HT *L. plantarum* CECT9161. An investigation of transcriptomic, metabolomic, and proteomic data would elucidate whether these differential gene abundances translate into functional differences.

Interestingly, the administration of a high dose of HT *L. plantarum* CECT9161 was associated with an increase in the abundance of genes connected with biofilm formation in dogs. This contrasts with the evidence that HT *L. plantarum* CECT9161 reduced biofilm formation in vitro and in the clinical trial. This discrepancy may be due to environmental conditions in vivo favouring the growth of microbial species that have an increased capacity for biofilm formation, without necessarily leading to functional changes that result in increased biofilm formation. For example, exposure of the in vivo biofilm to dynamic factors such as saliva flow, the mechanical action of chewing, and host immune response may select for bacterial species that possess a greater relative abundance of biofilm-associated genes. Investigating the expression of biofilm-associated genes and the production of biofilm-associated proteins would help elucidate whether these genes were expressed, and if so, how they affected biofilm dynamics.

A particular strength of the present study was in the principled selection of the oral health postbiotic using a rational selection of progressive preclinical assays, including microbial inhibition, the xCELLigence RTCA assay, and the inflammatory model using an oral epithelial cell line. Moreover, the use of shotgun metagenome sequencing of supragingival plaque in the clinical trial provided insights into the oral microbiome functional potential, rarely seen in canine clinical studies. However, there are limitations to these data. The duration of the clinical study was comparatively short in the context of oral health, which is typically understood in the period of years, rather than days. The study was conducted in a small and comparatively uniform population of research animals fed a single diet. An investigation of the postbiotic over a longer intervention period in a large-scale field trial of pet dogs, who have not undergone recent teeth cleaning, and who vary in age, breed, diet, and health status would establish the generalisability of the results obtained in the present trial. Furthermore, investigating the postbiotic in formats with an extended contact time, such as chewable tablets or dental chews, would elucidate whether increasing the contact time of the postbiotic increases its efficacy, as would studying the postbiotic alongside a wet diet that lacks the mechanical action of a kibble diet. Considering that the persistence of the postbiotic in saliva when administered as a top dressing on kibble is not known, future work should quantify the postbiotic in saliva when delivered in different formats to elucidate their effects on oral contact time.

Testing the postbiotic administered alongside a wet diet, which is expected to be associated with a lower level of mechanical action than kibble, may also provide the postbiotic a greater opportunity to exert an effect on plaque formation. Microbiome analysis was hindered by the relative immaturity of the canine oral microbiome field; current reference databases for the analysis of the oral microbiome in dogs are human-orientated and limited in scope. Frequently, up to 50% of sequences could not be classified, hindering the taxonomical classification of supragingival plaque samples. Furthermore, not all sequences in the databases available had a valid name or identification, so results relating to unnamed clades were omitted as they were uninterpretable. Although we observed changes in the metagenome, we did not investigate the functional consequences by assessing gene expression or protein production due to constraints on the resources available for the study. Future work should incorporate transcriptomic, proteomic, and metabolomic analysis to investigate changes in the functionality of the supragingival plaque microbiome. Finally, no baseline supragingival plaque samples were collected due to the teeth cleaning on Day 0, preventing within-subject comparisons to be made and consequently limiting the inference of a causal relationship from the microbiome changes. Future studies in dogs with established dental plaque biofilms and the presence of plaque and calculus are warranted. Moreover, the evaluation of HT *L. plantarum* CECT 9161 in a dental chew formulation to increase oral contact time and to combine the postbiotic with mechanical plaque disruption may offer another opportunity to positively modulate canine oral health.

## 5. Conclusions

The present study identified a novel postbiotic for oral health in dogs and investigated its effects on oral health scores in healthy dogs during the initial stages of biofilm and plaque formation following teeth cleaning. We revealed that postbiotic HT *L. plantarum* CECT 9161 may be associated with reduced plaque formation and the modulation of the microbiome of supragingival plaque following plaque removal in dogs. This provides the first evidence for a role of the postbiotic to support a healthy mouth in dogs. Further work should investigate the postbiotic in a diverse population of pet dogs, over a longer intervention period, and investigate the functional effects of the postbiotic on the oral microbiome by utilising transcriptomic, metabolomic, and proteomic analysis.

## Data Availability

Sequence files and metadata for the microbiome analyses have been deposited in NCBI SRA under BioProject PRJNA1150971.

## Supplementary Materials

The following supporting information can be downloaded at: https://www.mdpi.com/article/10.3390/ani15111615/s1. Figure S1: Complementary data obtained from preclinical assays. Figure S2: Change in halitosis, gingivitis, and calculus from baseline at Days 29 and 57. Figure S3: Diversity analysis of supragingival plaque microbial composition. Figure S4: BLASTn alignment of the genes against NCBI nt database. Figure S5: Significant gene enrichment of module M00529. Table S1: Summary statistics of baseline data for halitosis and gingivitis. Table S2: Summary statistics of the change in gingivitis from baseline by intervention and timepoint. Table S3: Predicted change in gingivitis by intervention group and timepoint estimated by the fixed-effects model. Table S4: Summary statistics of the change in halitosis from baseline by intervention and timepoint. Table S5: Predicted change in halitosis by intervention group and timepoint estimated by the fixed-effects model. Table S6: Summary statistics of the change in calculus from baseline by intervention and timepoint. Table S7: Predicted change in calculus by intervention group and timepoint estimated by the fixed-effects model. Table S8: Summary statistics of the change in plaque from baseline by intervention and timepoint. Table S9: Predicted change in plaque by intervention group and timepoint estimated by the fixed-effects model. Table S10: Probiotic strains used in the study. Supplementary Methods: Descriptions of preclinical methods, including probiotic preparation, DNA sequencing, biofilm characterization, and clinical protocols.
