# Supplementary material for: Postbiotic Lactiplantibacillus plantarum CECT 9161 Influences the Canine Oral Metagenome and Reduces Plaque Biofilm Formation
Source: Animals (Basel). 2025 May 30;15(11):1615. doi: 10.3390/ani15111615 (PMC12153843; doi:10.3390/ani15111615)
Supplement: Supplementary file 1 [file animals-15-01615-s001.zip › animals-3630906-supplementary.pdf]

Article

Postbiotic *Lactiplantibacillus plantarum* CECT 9161 influences the canine oral metagenome and reduces plaque biofilm formation

Supplementary Materials

Classification: Confidential

Supplementary Results

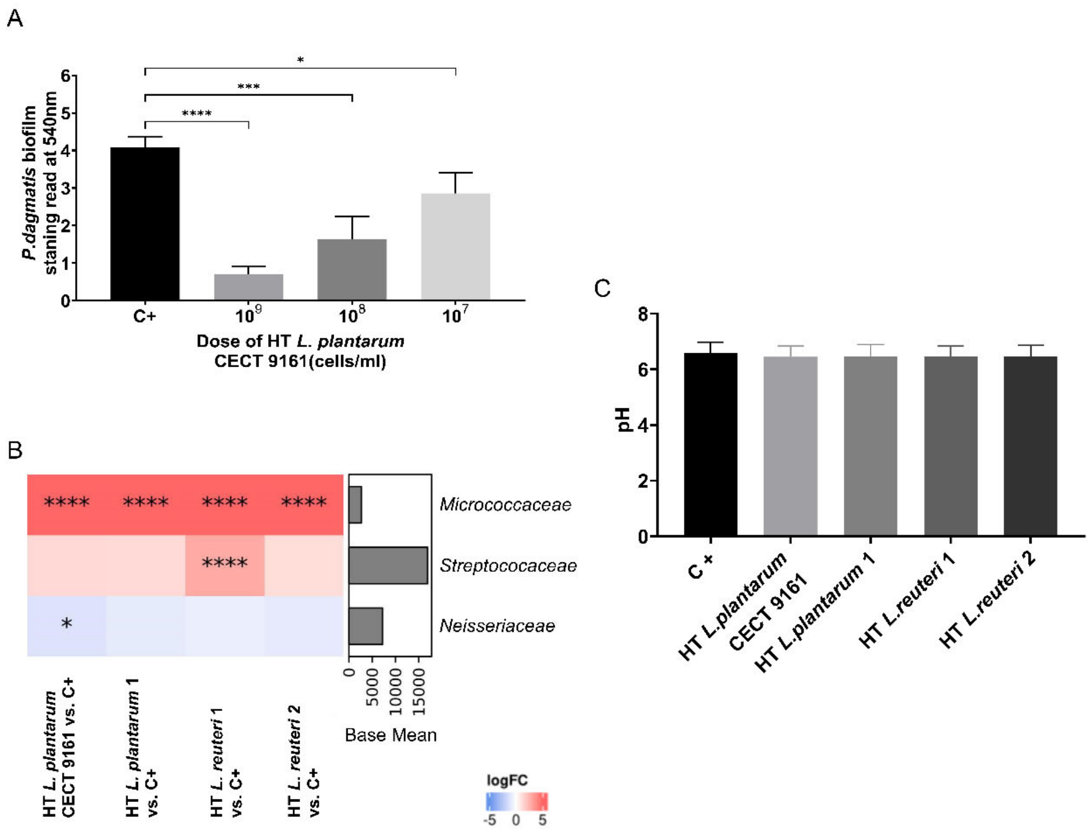

Figure S1. Complementary data obtained from preclinical assays.

(**A**) Biofilm inhibition of *P. dagmatis* ATCC 51570 when co-cultured with HT *L. plantarum* CECT 9161. There was a significant reduction at all doses tested. Statistical significance was tested in Graphpad Prism using a one-way ANOVA with Dunnett's test post-hoc analysis and $p < 0.05$ was deemed statistically significant. * $p < 0.05$, *** $p < 0.001$, **** $p < 0.0001$. (**B**) LogFC of the comparisons of family abundances between each postbiotic group against the control group (C+). Red on the heatmap indicates the bacteria had a higher abundance in the postbiotic than in the control and blue shows that the bacteria had a lower abundance. The taxa shown were present in ≥ 50% of the samples in ≥ 1 of the compared groups. Statistical analysis was tested in R with 'limma-voom' package using its internal moderated t-test. * adj. $p < 0.05$, **** $p < 0.0001$. (**C**) Influence of the postbiotics on pH of culture media; no significant differences were observed.

adj., adjusted; C+, control; DNA, deoxyribonucleic acid; HT *L. plantarum* CECT 9161; heat-treated *Lactiplantibacillus plantarum* CECT 9161; HT *L. plantarum* 1,

Classification: Confidential

heat-treated *Lactiplantibacillus plantarum* 1; HT *L. reuteri* 1; heat-treated *Lactiplantibacillus reuteri* 1; HT *L. reuteri* 2, heat-treated *Lactiplantibacillus reuteri* 2; logFC, log fold change; N, total number of dogs; *P. dagmatis*, *Pasteurella dagmatis* ATCC 51570; pH, potential of hydrogen; SD, standard deviation.

Table S1. Summary statistics of baseline data for halitosis and gingivitis.

**Parameter**	**Group**	**Mean**	**SD**	**n**
**Halitosis**	CON	124.80	28.00	20
	LOW	124.05	25.00	20
	HIGH	126.57	71.00	20
**Gingivitis**	CON	0.71	0.33	20
	LOW	0.66	0.29	20
	HIGH	0.65	0.25	20

CON, control group; HIGH, high dose heat-treated *Lactiplantibacillus plantarum* CECT 9161 group; LOW, low dose heat-treated *Lactiplantibacillus plantarum* CECT 9161 group; n, number of dogs in each intervention group; SD, standard deviation.

Table S2. Summary statistics of the change in gingivitis from baseline by intervention and timepoint.

**Timepoint**	**Group**	**Mean**	**Median**	**Min**	**Max**	**SD**	**n**
**Day 29**	CON	–0.09	–0.06	–0.39	0.11	0.13	20
	LOW	–0.07	–0.06	–0.39	0.22	0.16	20
	HIGH	0.02	0.00	–0.28	0.61	0.21	20
**Day 57**	CON	–0.01	0.00	–0.28	0.28	0.16	20

	LOW	–0.05	0.00	–0.33	0.11	0.13	20
	HIGH	0.01	–0.03	–0.28	0.83	0.26	20

CON, control group; HIGH, high dose heat-treated *Lactiplantibacillus plantarum* CECT 9161 group; LOW, low dose heat-treated *Lactiplantibacillus plantarum* CECT 9161 group; Max, maximum; Min, minimum; n, number of dogs in each intervention group; SD, standard deviation.

Table S3. Predicted change in gingivitis by intervention group and timepoint estimated by the fixed-effects model.

**Timepoint**	**Group**	**Estimated effect**	**95% CI**
**Day 29**	CON	–0.05	(–0.12, 0.02)
	LOW	–0.08	(–0.15, 0.00)
	HIGH	–0.01	(–0.07, 0.06)
**Day 57**	CON	–0.03	(–0.10, 0.04)
	LOW	–0.05	(–0.12, 0.02)
	HIGH	0.02	(–0.05, 0.09)

CI, confidence interval; CON, control group; HIGH, high dose heat-treated *Lactiplantibacillus plantarum* CECT 9161 group; LOW, low dose heat-treated *Lactiplantibacillus plantarum* CECT 9161 group.

T**able S4. Summary statistics of the change in halitosis from baseline by intervention and timepoint.**

**Timepoint**	**Group**	**Mean**	**Median**	**Min**	**Max**	**SD**	**n**
**Day 29**	CON	31.27	30.33	–39.67	91.33	28.98	20

	LOW	26.53	27.67	–44.00	81.00	31.52	20
	HIGH	52.12	50.67	–21.00	200.67	49.59	20
**Day 57**	CON	10.75	11.83	–40.33	80.00	26.87	20
	LOW	8.61	12.00	–50.67	74.00	32.63	20
	HIGH	37.63	21.00	–23.67	352.33	80.46	20

CON, control group; HIGH, high dose heat-treated *Lactiplantibacillus plantarum* CECT 9161 group; LOW, low dose heat-treated *Lactiplantibacillus plantarum* CECT 9161 group; Max, maximum; Min, minimum; n, number of dogs in each intervention group; SD, standard deviation.

Table S5. Predicted change in halitosis by intervention group and timepoint estimated by the fixed-effects model.

Timepoint	Group	Estimated effect	95% CI
**Day 29**	CON	31.87	(20.32, 43.41)
	LOW	28.56	(16.75, 40.37)
	HIGH	41.29	(29.45, 53.12)
**Day 57**	CON	11.54	(–0.01, 23.09)
	LOW	8.24	(–3.58, 20.05)
	HIGH	20.96	(9.14, 32.79)

CI, confidence interval; CON, control group; HIGH, high dose heat-treated *Lactiplantibacillus plantarum* CECT 9161 group; LOW, low dose heat-treated *Lactiplantibacillus plantarum* CECT 9161 group.

Classification: Confidential

Table S6. Summary statistics of the change in calculus from baseline by intervention and timepoint.

Timepoint	Group	Mean	Median	Min	Max	SD	n
**Day 29**	CON	1.59	1.64	0.00	3.22	0.78	20
	LOW	1.14	0.89	0.39	2.67	0.64	20
	HIGH	1.57	1.39	0.22	3.50	0.81	20
**Day 57**	CON	1.98	2.06	0.28	3.11	0.84	20
	LOW	1.63	1.33	0.61	3.67	0.75	20
	HIGH	1.83	1.72	0.33	3.33	0.84	20

CON, control group; HIGH, high dose heat-treated *Lactiplantibacillus plantarum* CECT 9161 group; LOW, low dose heat-treated *Lactiplantibacillus plantarum* CECT 9161 group; Max, maximum; Min, minimum; n, number of dogs in each intervention group; SD, standard deviation.

Table S7. Predicted change in calculus by intervention group and timepoint estimated by the fixed-effects model.

Timepoint	Group	Estimated effect	95% CI
**Day 29**	CON	1.60	(1.25, 1.94)
	LOW	1.20	(0.86, 1.54)
	HIGH	1.51	(1.17, 1.85)
**Day 57**	CON	1.97	(1.63, 2.31)
	LOW	1.57	(1.23, 1.85)
	HIGH	1.89	(1.54, 2.23)

CI, confidence interval; CON, control group; HIGH, high dose heat-treated *Lactiplantibacillus plantarum* CECT 9161 group; LOW, low dose heat-treated *Lactiplantibacillus plantarum* CECT 9161 group.

Table S8. Summary statistics of the change in plaque from baseline by intervention and timepoint.

Timepoint	Group	Mean	Median	Min	Max	SD	n
**Day 29**	CON	4.53	4.67	1.56	7.56	1.46	20
	LOW	3.76	3.33	1.78	6.11	1.21	20
	HIGH	4.68	4.69	2.06	7.17	1.32	20
**Day 57**	CON	4.92	4.86	1.94	7.67	1.39	20
	LOW	4.04	3.89	1.94	6.28	1.07	20
	HIGH	4.21	4.11	1.78	7.56	1.46	20

CON, control group; HIGH, high dose heat-treated *Lactiplantibacillus plantarum* CECT 9161 group; LOW, low dose heat-treated *Lactiplantibacillus plantarum* CECT 9161 group; Max, maximum; Min, minimum; n, number of dogs in each intervention group; SD, standard deviation.

Table S9. Predicted change in plaque by intervention group and timepoint estimated by the fixed-effects model.

Timepoint	Group	Estimated effect	95% CI
**Day 29**	CON	4.53	(3.94, 5.12)
	LOW	3.76	(3.17, 4.35)
	HIGH	4.68	(4.09, 5.27)

**Day 57**	CON	4.92	(4.32, 5.51)
	LOW	4.04	(3.46, 4.63)
	HIGH	4.21	(3.62, 4.80)

CI, confidence interval; CON, control group; HIGH, high dose heat-treated *Lactiplantibacillus plantarum* CECT 9161 group; LOW, low dose heat-treated *Lactiplantibacillus plantarum* CECT 9161 group.

Classification: Confidential

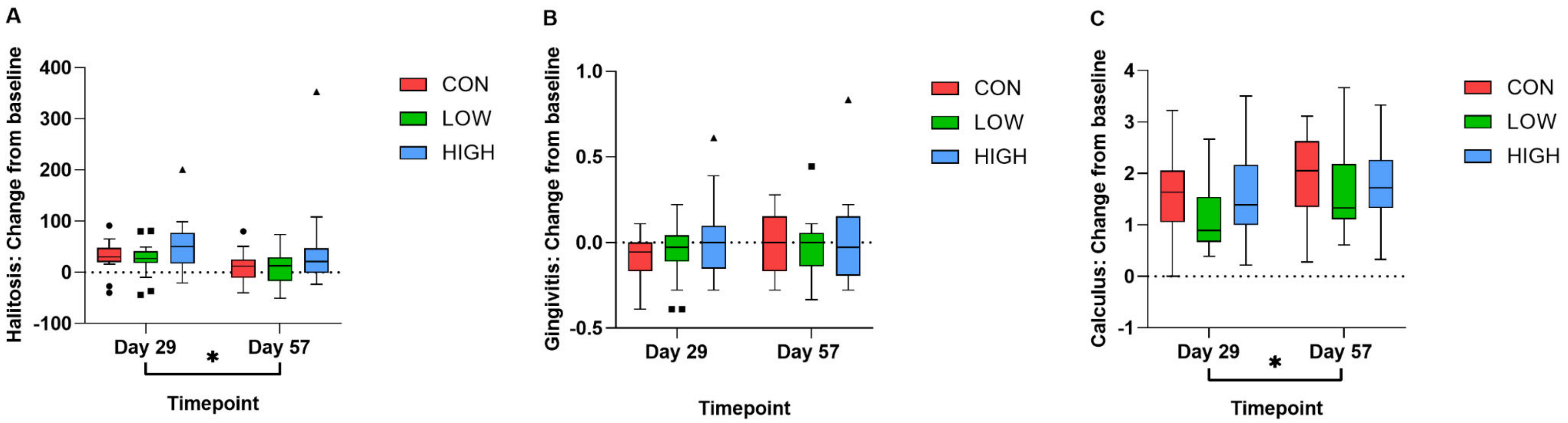

Figure S2. Change in halitosis, gingivitis, and calculus from baseline at Days 29 and 57.

Dogs (N = 60) were stratified into three intervention groups (CON [n = 20], LOW [n = 20] and HIGH [n = 20]). Boxplots show the change in halitosis (**A**), gingivitis (**B**), and calculus (**C**) scores from baseline in each intervention group at Days 29 and 57. The box includes the upper and lower quartiles and, therefore, the middle 50% of the data and the horizonal line within the box are the median. The whiskers extend to 1.5 times the interquartile range and data points outside of this range are marked as squares (LOW) or triangles (HIGH). No statistically significant intervention effect was observed; however, there was a significant decrease in halitosis score across all groups at Day 57 compared with Day 29, and calculus was significantly increased in all groups at Day 57 compared with Day 29. Statistical significance was tested using a mixed-effects linear model and $p < 0.05$ was deemed statistically significant. * $p < 0.05$.

CON, control group; HIGH, high dose heat-treated *Lactiplantibacillus plantarum* CECT 9161 group; LOW, low dose heat-treated *Lactiplantibacillus plantarum* CECT 9161 group; N, total number of dogs; n, number of dogs in each intervention group.

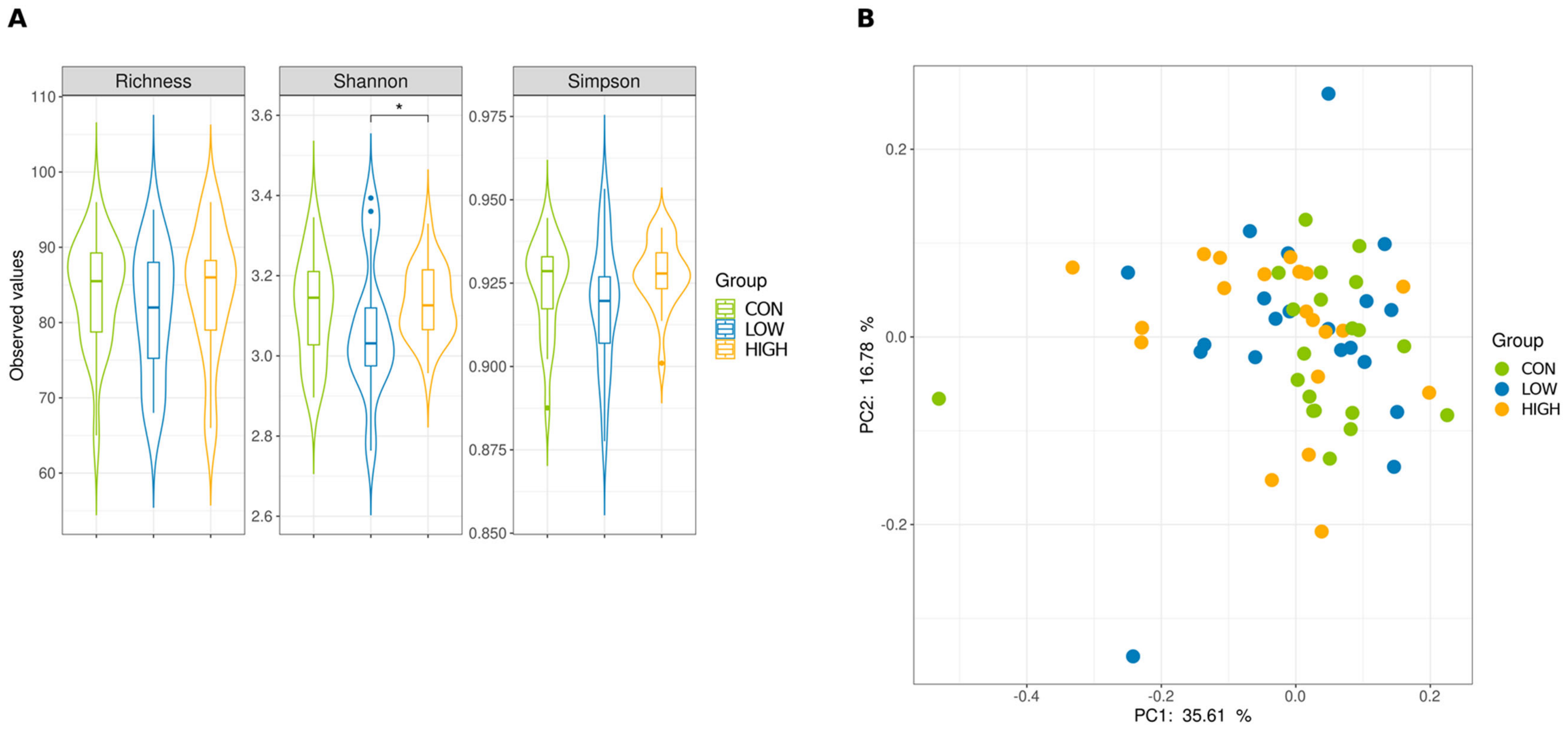

Figure S3. Diversity analysis of supragingival plaque microbial composition.

(**A**) Boxplots of Richness, Shannon, and Simpson indexes of each intervention group and (**B**) PCoA presentation of samples at species level for each intervention group. Significant increases in Shannon alpha diversity metric were observed in the HIGH group when compared with the LOW group. PCoA at species level suggested no evidence of clustering of the samples according to intervention group. Statistical significance was tested in R with ‘stats’ package using Wilcoxon signed-rank test. * $p < 0.05$.

CON, control group; HIGH, high dose heat-treated *Lactiplantibacillus plantarum* CECT 9161 group; LOW, low dose heat-treated *Lactiplantibacillus plantarum* CECT 9161 group; PC, principal component; PCoA, principal coordinate analysis.

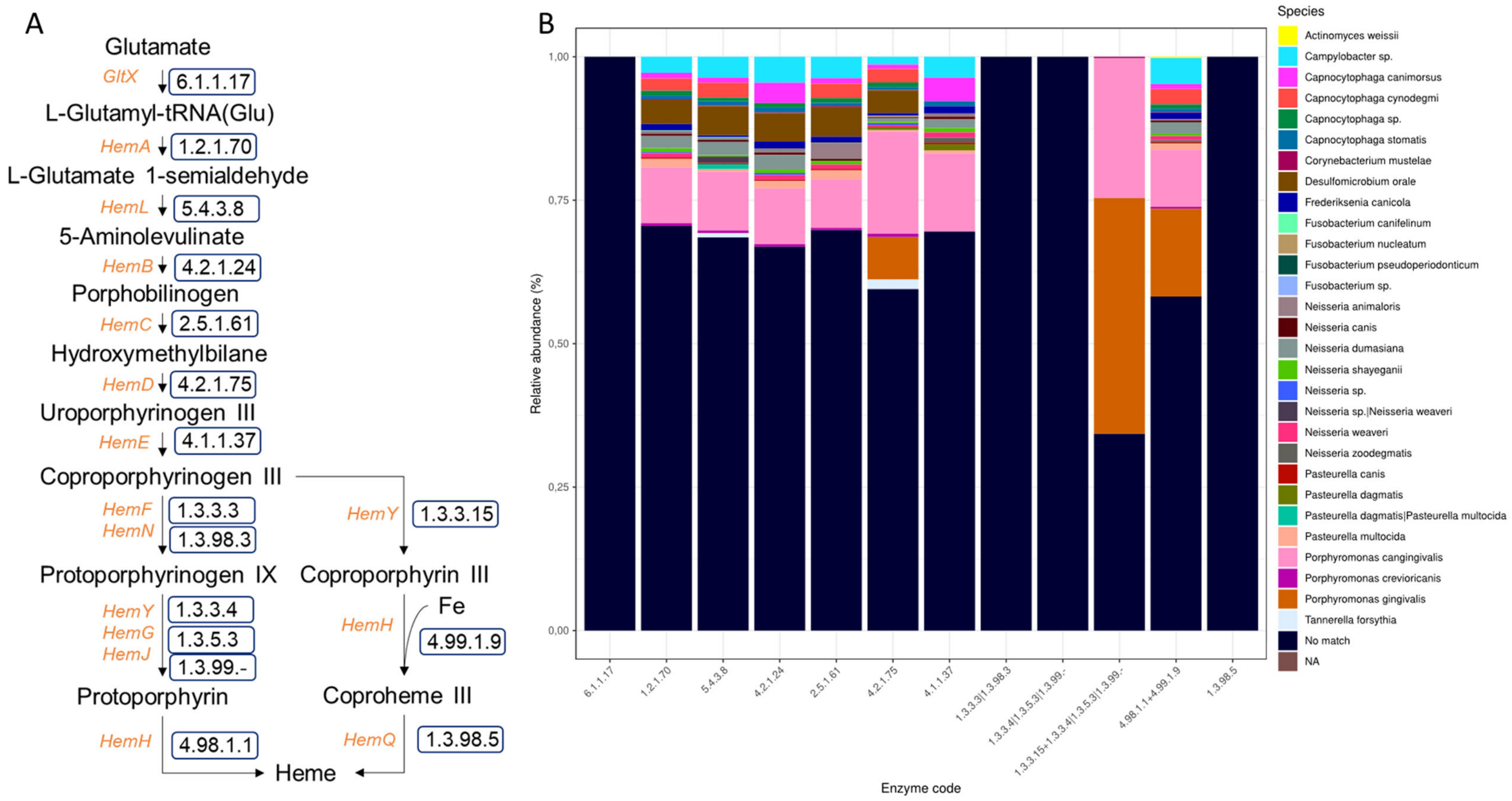

Figure S4. BLASTn alignment of the genes against NCBI nt database.

(**A**) Heme biosynthesis modules M00926 and M00121 pathways from the KEGG database. (**B**) Relative abundances of each enzyme colored by its taxonomic annotations at species level obtained by the BLASTn of the gene sequences against NCBI nt database. Genes that had a match were mostly taxonomically classified as *Porphyromonas cangingivalis* and *Porphyromonas gingivalis*.

NCBI, National Center for Biotechnology Information; nt, nucleotide.

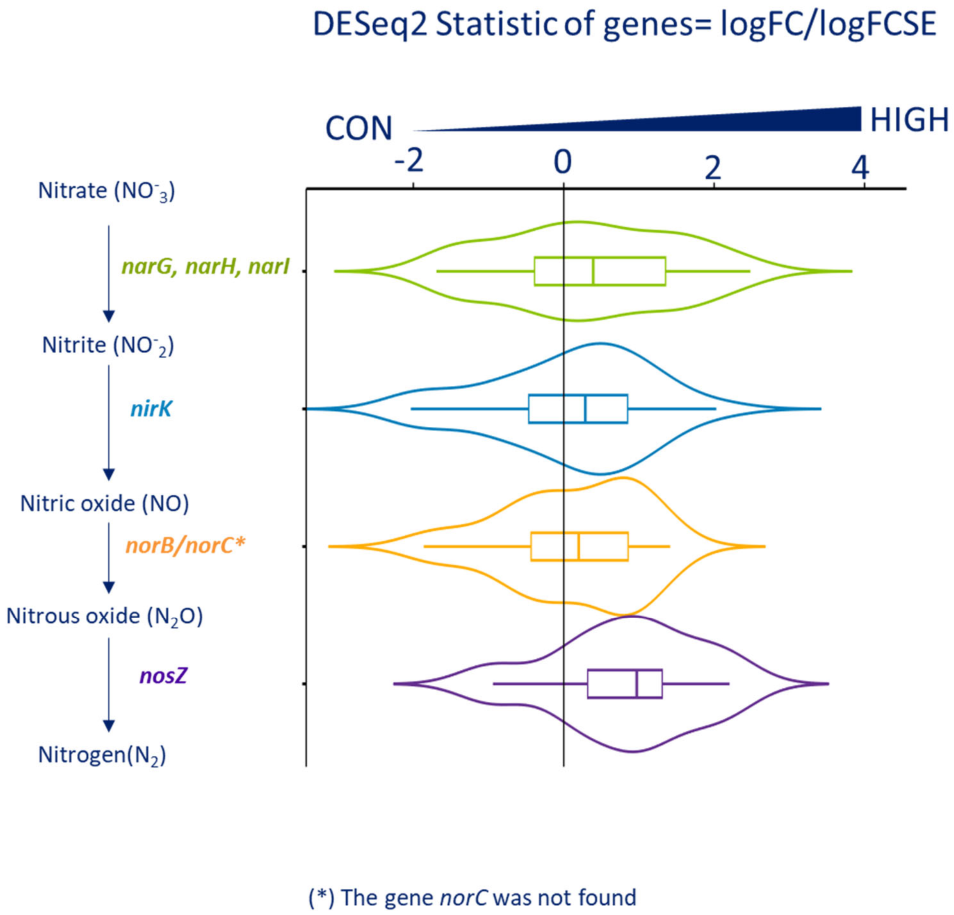

Figure S5. Significant gene enrichment of module M00529.

Left: Schematic of module M00529 denitrification. **Right:** Violin boxplots showing the stat from DESeq2 on each set of genes. Stat is calculated by the log2FC divided by its standard error and it is used to rank the genes for the GSEA. M00529 (denitrification) was significantly enriched in the HIGH group compared with the CON group.

CON, control group; GSEA, gene set enrichment analysis; HIGH, high dose heat-treated *Lactiplantibacillus plantarum* CECT 9161 group; logFC, log fold change.

Supplementary Methods

Preclinical phase

Probiotic and postbiotic preparation

Table S10. Probiotic strains used in the study.

Probiotic/postbiotic strain	Species	Origin
Lactiplantibacillus plantarum CECT 9161	*Lactiplantibacillus* sp.	Human oral sample
HT *Lactiplantibacillus plantarum* 1	*Lactiplantibacillus* sp.	Human sample
HT *Lactiplantibacillus reuteri* 1	*Lactiplantibacillus* sp.	Canine sample
HT *Lactiplantibacillus reuteri 2*	*Lactiplantibacillus* sp.	Canine sample

HT, heat-treated; sp, species.

Flow cytometry analysis

The CytoFlex cytometer was programmed for use with the following settings:

- Laser: 488 nm
- Filters: FITC (525/40 BP), PE (585/42 BP), ECD (610/20 BP), PC5.5 (690/50 BP), and PC7 (780/60 BP)
- Gain: Forward side scatter 1000, Side scatter 400, FITC 240, PE 180 and PC5.5 470
- The samples were diluted 1 in 10000 and run twice.

Growth inhibition of canine oral early colonizers

Samples from the gingiva, teeth and palate were collected from 6 healthy dogs. All samples were cultivated in MRS with 0.05% g/L cysteine, BHI broth (Oxoid, Hampshire, UK) and blood agar media under aerobic and anaerobic conditions. After 48 hours of incubation different colonies were isolated and identified by 16S rRNA gene sequencing. Strains isolated included canine oral early colonizers; *P. dagmatis* ATCC 51570, *N. zoodegmatis* and *N. weaveri* [8]. The early-colonizer strains were

cultured in BHI broth (Oxoid, Hampshire, UK) under aerobic conditions at 37 ºC for 24 hours.

DNA isolation and amplicon sequencing of biofilm samples

DNA was isolated using the MagNA Pure LC 2.0 Instrument and MagNA Pure LC DNA Isolation Kit III for Bacteria and Fungi (Roche Diagnostics, Indianapolis, IN, USA) and quantified by Qubit 3 Fluorometer (Thermo Fisher Scientific, Waltham, MA, USA) in accordance with the manufacturer's instructions. A total of 5 ng DNA from biofilm samples was amplified following the 16S Metagenomic Sequencing Library Illumina 15044223 B protocol (Illumina, San Diego, CA, USA). Library quality control and normalization were ensured by profiling and length distribution analysis using the HSD5000 kit in the TapeStation 4200 equipment (Agilent, Santa Clara, CA, USA) and quantitation with Qubit dsDNA Quantification Assay Kits (Thermo Fisher Scientific, Waltham, MA, USA). Miseq sequencing platform in a 300 paired-end reads configuration generated *.fatq files as processed sequencing output (Miseq Control software version 2.6). Details of the preprocessing metagenomic analysis are described in the subsequent **Preprocessing metagenomic analysis** section below.

Raw sequences, forward and reverse, were merged to obtain the complete sequence using the BBMerge package from BBMap V.38 software [51]. The amplification primers from the sequences obtained in the sequencing step were trimmed with 'Cutadapt v 1.8.1' and parameters by default [52] and sequences < 200 nts were removed from the analysis. A quality filter was applied to delete poor-quality sequences using Reformat module from BBMap V.38 software. Sequences with < 200 nts were removed and those bases in extreme positions that did not reach Q20 or greater phred score were also removed. Subsequently, sequences whose average quality did not surpass the Q20 threshold as a mean quality of the whole sequence were also deleted.

The reads were processed using the DADA2 [53] denoise-single command. Error rates were learned from a set of subsampled reads using 'learnErrors' and a sample inference algorithm was applied with the 'dada' function to generate ASVs. The chimeric ASVs were removed using 'removeChimeraDenovo'. Those clean ASVs were annotated against the NCBI 16S rRNA database version 2022 using BLASTn

version 2.2.29+ [54]. The taxonomy of the ASVs that had been assigned with a lower percentage identity than 97% was reassigned using NBAYES algorithm [55]. NBAYES classifier was trained on V3-V4 regions of 16S rRNA gene from SILVA v.138 database [56].

Characterization of *in vitro* microbiota biofilm

The feature table was normalized using calcNormFactors function, with the trimmed mean of M-values (TMM) option. After normalization the limma [57] (v.3.42.2) function voom was used to convert normalized counts to log2-counts-per-million and assign precision weights to each observation based on the mean-variance trend. The functions lmFit, eBayes, and topTable in the limma R package were used to fit weighted linear regression models, perform tests based on an empirical Bayes moderated t-statistic and to obtain BH false discovery rate (FDR)-corrected p values. A taxon was considered differentially abundant if $p < 0.05$ and if it was present in ≥ 50% of the samples of one of the compared groups. The linear model included the subject variable as a random effect. Heatmaps were constructed using ComplexHeatmap R package v.2.11.1 [50].

Clinical phase

Animal husbandry

Dogs were housed individually for feeding and sample collection only and, for the remaining time, pair- or group-housed. The dogs had group access to outdoor runs for a minimum of 1 hour per day and visited a large grassy paddock at least twice weekly for robust play and exercise. Ventilation was provided by use of an air exchange unit and the temperature and humidity level was maintained at a comfortable level. All dogs were fed a standard diet of Purina Dog Chow (a kibble, fed dry) topped with either the control placebo powder (CON) or one of two test supplement powders (LOW or HIGH) for the duration of the study (57 days). Fresh, clean water was available *ad libitum.* The quantity of food provided to each dog was adjusted to maintain body weight, and consumption of food was measured and recorded daily for each dog. The number of animals required was based on detecting a mean difference of 15% reduction in plaque score.

Pre-test phase

A pre-test phase (Day –7) was conducted before the initiation of the 57-day trial. During this period, dogs were weighed and fed the standard diet once daily. Each dog had their teeth scaled with an ultrasonic scaler and polished with a glycerine/pumice paste under general anesthesia on Day –7 and Day 0. Dogs were stratified using the plaque scores taken on Day 0 and they were divided into three groups (Group A, Group B, and Group C). Each group had a similar level of variation of plaque. Each animal was given a study identity as Dog 1, Dog 2, etc. in alphabetical, consecutive order.

The dogs were premedicated and anesthetized for tooth scaling and polishing according to the following procedure:

- Premedication: 2 ml butorphanol (10 mg/ml), 0.3 ml acepromazine (10 mg/ml) and 8 ml atropine (0.5 mg/ml) given at 0.03 ml/kg
- Induction: Propofol (10 mg/ml) given at 5.5 mg/kg intravenously
- Maintenance: 2% isoflurane and oxygen
- Dogs were placed on intravenous fluids (Plasmalyte A) for all procedures involving anesthesia.

After cleaning and polishing, teeth were disclosed with a 2% eosin solution and an oral examination was performed to ensure there was no remaining plaque or calculus build up, resulting in a clean mouth model.
